# 24-48 h initiation by transdermal buprenorphine for the treatment of opioid use disorder in the inpatient setting: a retrospective chart review

**DOI:** 10.1186/s13722-026-00657-3

**Published:** 2026-03-13

**Authors:** Pouya Azar, Victor W. Li, Mohammadali Nikoo, Jennifer LaBranche, James S. H. Wong, Jessica Machado, Tam To, Alaa Al Hawamdeh, Martha J. Ignaszewski, Andrew A. Herring, Marc Vogel, Laura Kehoe, Amer Raheemullah, Ruchi Fitzgerald, Reinhard M. Krausz, Julio S. G. Montaner, Anil R. Maharaj

**Affiliations:** 1 Complex Pain and Addiction Services, Department of Psychiatry, Vancouver Coastal Health, Vancouver, BC Canada; 2 Department of Psychiatry, Faculty of Medicine, University of British Columbia, Vancouver, BC Canada; 3 Children’s and Adolescent Psychiatry, BC Children’s Hospital, Vancouver, BC Canada; 4 Highland General Hospital, Alameda Health System, Oakland, CA USA; 5 Department of Emergency Medicine, University of California, San Francisco, CA USA; 6 Massachusetts General Hospital, Department of Internal Medicine, Boston, MA USA; 7 Harvard Medical School, Boston, MA USA; 8 Department of Psychiatry and Behavioral Sciences, Stanford University School of Medicine, Stanford, California USA; 9 Rush University, Chicago, IL USA; 10 British Columbia Centre for Excellence in HIV/AIDS, Providence Health Care, University of British Columbia, Vancouver, Canada; 11 Faculty of Pharmaceutical Sciences, University of British Columbia, Vancouver, British Columbia, Canada; 12 Division of Addictive Disorders, University of Basel Psychiatric Clinics, Wilhelm Klein-Strasse 27, Basel, 4012 Switzerland

**Keywords:** Opioid use disorder, Fentanyl, Buprenorphine, Transdermal, Initiation, Buprenorphine extended-release, Withdrawal, Opioid agonist treatment, Medications for opioid use disorder

## Abstract

**Background:**

Buprenorphine (Bup) is a first-line medication for opioid use disorder (OUD) that carries risk of precipitated withdrawal (PW) with ongoing need for rapid and effective initiation strategies. We report a novel approach to low-dose initiation using transdermal Bup patches (TD-Bup) and characterize the efficacy and tolerability of 24 h and 48 h TD-Bup initiation protocols for Bup as a medication for OUD (MOUD).

**Methods:**

We conducted a retrospective chart review using data extracted from paper and electronic medical records of adult inpatients with OUD admitted to Vancouver General Hospital between January 2022 and October 2023. Eligible patients were started on low-dose transdermal buprenorphine (TD-Bup) initiation protocols. The intervention involved sequenced application of TD-Bup patches reaching a total of 12 patches (240 µg/h) over 24 to 48 hours, with continuation of full opioid agonists. Protocol variants included: 6 patches q24h for 48 hours, 6 patches q12h for 24 hours, and 4 patches q8h for 24 hours. Primary outcomes were the rate of successful initiation onto therapeutic doses of sublingual or extended-release depot Bup, and the incidence of opioid withdrawal.

**Results:**

Seventy five patients were identified. Fifty five (73%) were successfully initiated and discharged on Bup and the most common reasons for unsuccessful initiation were preference to switch to another medication for OUD (*n* = 11) and withdrawal (*n* = 6). There was no significant difference between the protocol variants for successful initiation (*p* = 0.191). Adverse events (agitation, increased pain, insomnia, pruritus) were reported in 5 dropouts. Only 28 patients had sufficient Clinical Opioid Withdrawal Scale (COWS) scores to allow for objective assessment of withdrawal, with the remainder evaluated from full chart review. Nineteen patients had COWS score elevation > 6 and/or any narrative documentation of withdrawal. Three patients were felt to represent probable PW. No relationships were found between withdrawals and protocol variant (*p* = 0.621) or successful initiation (*p* = 0.082). All-cause withdrawals were associated with self-directed discharges (*p* = 0.042).

**Conclusions and relevance:**

Rapid low-dose buprenorphine initiation with TD-Bup appears feasible with relatively low risk of withdrawals and PW, although a weakness is the vulnerability to underdosing patients with full opioid agonists resulting in opioid deficit withdrawal. Further prospective evaluation of d rapid low-dose buprenorphine initiation with TD-Bup against other evidence-based contemporary Bup MOUD initiations is warranted.

**Supplementary Information:**

The online version contains supplementary material available at 10.1186/s13722-026-00657-3.

## Background

The opioid epidemic remains a crisis in North America, with overdose deaths increasing tenfold in the last two decades [1]. Medication for opioid use disorder (MOUD) is the mainstay in opioid use disorder (OUD) for treatment and overdose protection.

Buprenorphine (Bup) is a first-line MOUD with a superior safety profile to alternatives in many clinical scenarios. Bup is a partial agonist at the µ-opioid receptor which confers a ceiling effect for respiratory depression and oversedation, and it has minimal effects on QTc prolongation [2, 3]. However, Bup may cause precipitated withdrawal (PW) in highly tolerant individuals with ongoing use of full agonist opioids [4], which often represents a barrier to its clinical use. Contemporary approaches now include high-dose and low-dose initiations. With high-dose initiations, patients using opioids who present in mild withdrawal (Clinical Opiate Withdrawal Scale (COWS) [5] score > 8) can immediately be started on therapeutic doses of sublingual Bup/naloxone (SL-Bup) with low risk for PW [6], with consideration made for avoiding oversedation or other toxic effects in individuals with low tolerance [6]. With low-dose initiations, PW is avoided by gradually titrating Bup in small increments over several days [7, 8]; as pioneered by the foundational Bernese method [9]. Low-dose initiations remain important in clinical care for patients who present seeking treatment while not yet in withdrawal and wishing to avoid withdrawal, but the shortest common protocols still require at least 3 days [10, 11]. This can be prohibitive for short stays in hospitalized settings, especially for individuals without stable housing and difficulties with self-managing an outpatient initiation plan.

Here, we have iterated on low-dose initiations by using TD-Bup instead of SL-Bup. This approach was guided by pharmacokinetic modeling to recreate serum levels consistent with our prior 48 h SL-Bup initiation protocol [12] and takes advantage of the sustained release to achieve faster initiations. We have previously published several case reports using TD-Bup initiation onto full-dose Bup-SL or extended-release depot Bup (Bup-XR) MOUD [10, 13, 14]. The purpose of this retrospective case series is to further characterize the effectiveness, tolerability, and safety of this approach.

## Methods

The study protocol was approved by the Clinical Research Ethics Board at the University of British Columbia (H23-02262).

### Study population

Guided by a Vancouver Coastal Health pharmacist and a research advisor from the Enterprise Data Governance Office, all inpatient admissions during January 1, 2022, to October 31, 2023 with TD-Bup on the medication administration record were identified. We excluded admissions using TD-Bup outside of a Bup initiation protocol, which were those using TD-Bup for pain management. Four patients previously reported in case reports [10, 13, 14] were included.

**Fig. 1:**
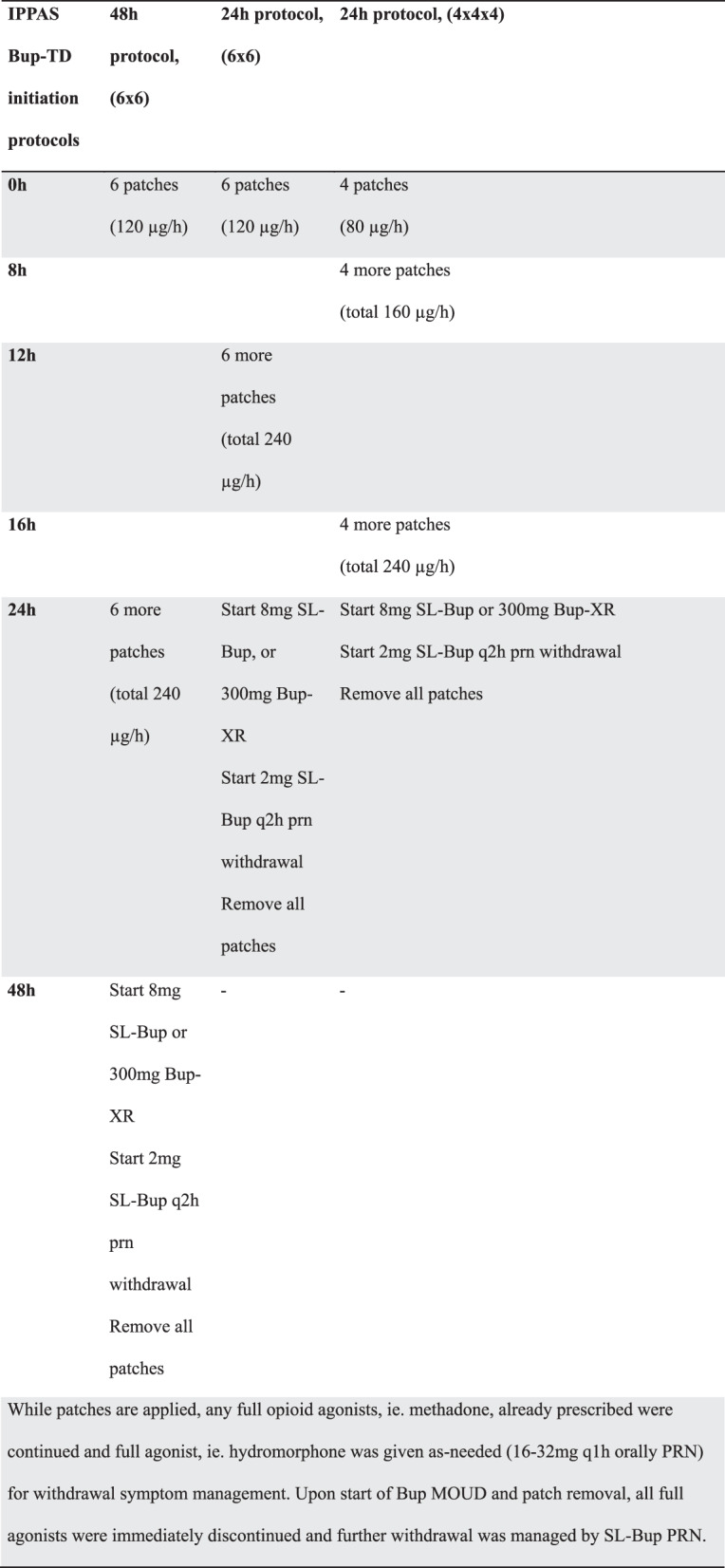

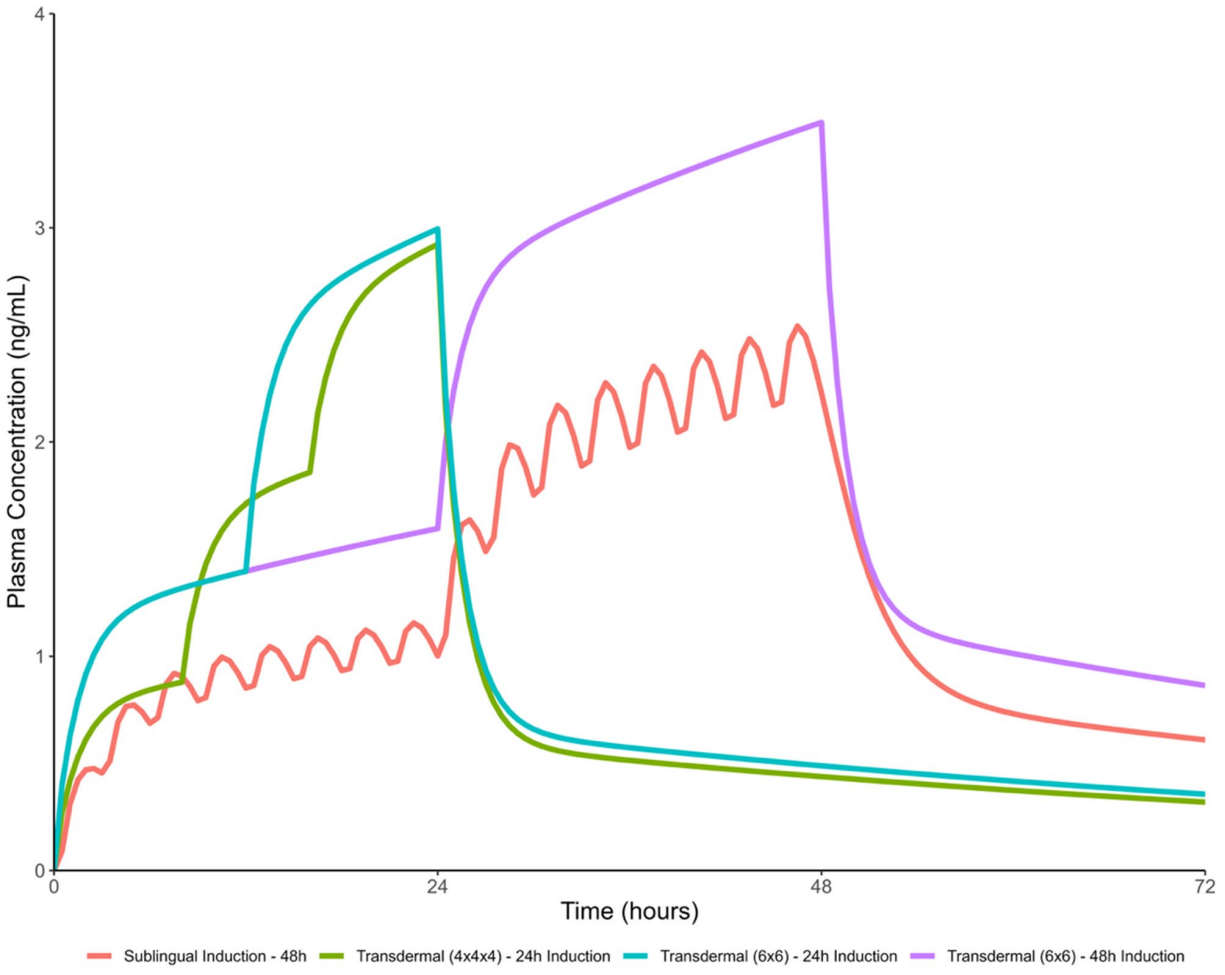
IPPAS TD-Bup initiation protocols, with pharmacokinetic modeling. **a**. Timeline of medication administrations for each protocol. **b**. These protocols are informed by pharmacokinetic modeling done by AM. Shown in the pink tracing is the projected serum levels using 0.5 mg bup sublingual film q3h for 24 h, followed by 1 mg q3h for 24 h, as previously reported [12, 15]. Overlaid traces for projected serum levels for the transdermal buprenorphine (TD-Bup protocols) are shown in comparison. Simulations were generated based on a previously published population pharmacokinetic modeling [16] and were used to guide the development of the TD-Bup protocols outlines in (**a**). Pharmacokinetic modelling was performed in R (v 4.2.3; R foundation for statistical computing, Vienna, Austria) using the rxode2 package [17]

Three Integrated Psychiatry Pain and Addiction (IPPAS) TD-Bup initiation protocols as previously described [13, 14] were included for this study (Fig. 1). The choice of initiation protocol changed over time, favoring simpler, faster variants given no perceived differences in outcomes at the time. Initially, almost all patients received the 48 h protocol (q24h application), with some receiving the 24 h twice-dosed protocol (q12h application). The 24 h thrice-dosed protocol (q8h application) was then briefly used, and subsequently, all patients exclusively received the 24 h twice-dosed protocol (q12h). In cases where patients were already on scheduled full agonist opioids (such as methadone), these were continued at full dose without taper throughout the initiation. All patients also had PRN oral hydromorphone to meet opioid requirements during the initiation. Scheduled and PRN full opioid agonists were immediately discontinued after administering SL-Bup or Bup-XR. Some patients required prescribed IV fentanyl on admission for very high opioid tolerances before the TD-Bup protocols were started, which is a method previously used for severe opioid withdrawal [18]. In these cases, the IV fentanyl was continued through the TD-Bup initiation process to serve as the full agonist PRNs, then discontinued after successful Bup initiation.

Data were abstracted from the now-retired mixed paper and electronic medical charting system in use at the time from January to November 2022, and subsequently from the modern fully paper-less electronic medical record (EMR), after its roll-out during this period. Data extraction was carried out by research assistants overseen by TT, AAH, and JSHW.

### Outcomes

Two primary outcomes were included: 1) rates of successful initiation onto Bup MOUD, and 2) whether opioid withdrawals, especially precipitated withdrawals, occurred during the initiation. Initiation was considered successful if the patient was started on therapeutic doses of SL-Bup or a Bup-XR injection (daily SL-Bup > 8 mg a day, Bup-XR 100 mg or 300 mg, based on the suggested therapeutic dose in the product monograph, and as a baseline upon which to titrate further without significant risk of PW). Because buprenorphine could technically be initiated “successfully” by causing precipitated withdrawal with a single therapeutic dose, the second primary outcome of withdrawals was important to determine efficacy. Withdrawal symptoms were assessed by repeated COWS assessments in the 24 h period prior to initiation, during initiation, and in the 24 h period after initiation, with particular focus on periods 1 h pre- and post- patch application or Bup MOUD start, given that the rate of rise for serum level is highest immediately after patch application according to PK modeling (Fig. 1). Withdrawals were defined as a COWS score change of > 6 [19, 20]. These were further defined as PW using a priori criteria: 1) a marked and rapid elevation in COWS score that occurs shortly (time < 1 hr) after administration of Bup or if significant changes in COWS scores were flagged outside of 1 hr; 2) minimal response to full agonist PRNs or response only at significantly higher doses than their previously demonstrated PRN usage; 3) not a result of clear deficit in full agonist relative to baseline use. All cases with withdrawals, and those missing adequate number of COWS scores for assessment (at least 1 COWS score between each intervention point), were flagged for independent full chart review by two expert clinicians (from VWL, MN, JM, MI) to discern if they met the above criteria for PW, with discrepancies resolved by a third clinician (PA).

Secondary outcomes included total dose of full agonist opioid used over the course of the protocol and rates of protocol deviations (patch application timings missed by >3 h, or Bup MOUD initiation missed by >1 h from patch removals). The latter was tracked and used to determine if they had any impact on the primary outcomes. Adverse events were tracked, as well as dropouts and associated reasons.

### Statistical analysis

Characteristics are summarized as medians (interquartile interval) or means (standard deviation) for measures with or without large skew, respectively. Categorical comparisons were by Fisher’s exact test. Repeated measures ANOVA was used to determine differences in required opioids over time. Calculations of total opioid doses to oral morphine equivalents (OMEs) were done by existing conversion tables [21–23]. Box-and-whisker plots for corresponding opioid usage for these patients were generated in R (v 4.3.3; R Foundation for Statistical Computing, Vienna, Austria) using the ggplot2 package [24].

## Results

### Patient demographic characteristics

**Fig. 2:**
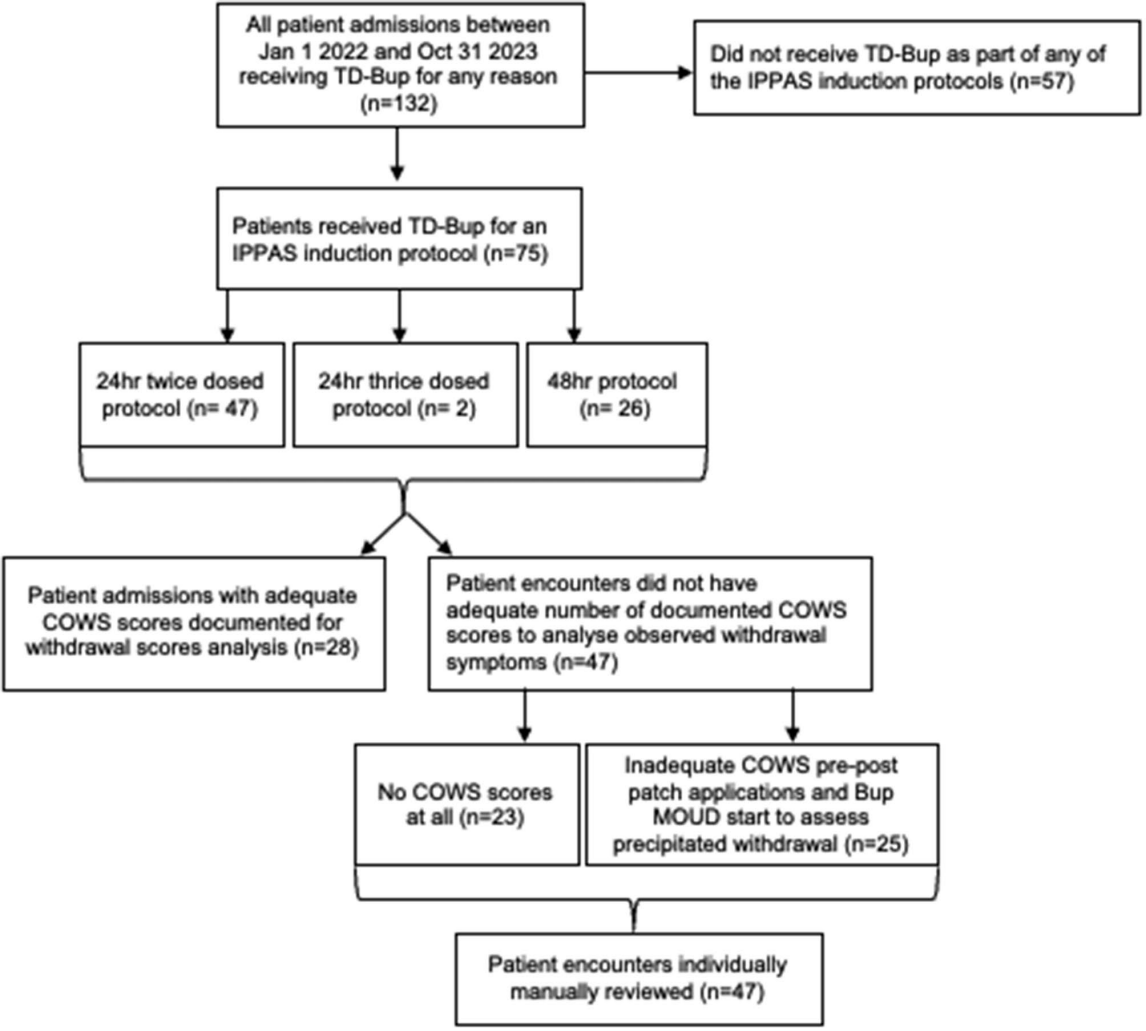
Flow diagram for chart retrieval and inclusion for retrospective chart review

A total of 132 admissions (30 on paper charts, 102 on the new paperless EMR) were identified (Fig. 2). Of these, 75 patients received a TD Bup initiation protocol: 26 patients received the 48 h protocol, 47 patients received the 24 h twice-dosed protocol and 2 patients received the 24 h thrice-dosed protocol. While most patients had tried MOUD at some point in their lives (66/75, 88%), only 33 patients had an active MOUD prescription of any dose at the time of admission and no patients were taking any form of Bup. Full demographic characteristics are shown in Table 1.

**Table 1:** Demographic characteristics (*n* = 75)

	No. (%) unless otherwise indicated
Age (Mean ((SD))	41.5 ± 11
Female	26 (35)
No stable living arrangement at time of admission	49 (65)
Duration of hospital stay (Median (IQR))	8 (4–16.5)
**Substance History**	
Opioid Use Disorder Diagnosis	75 (100)
Mild	3 (4)
Moderate	3 (4)
Severe	31 (41)
Not specified	38 (51)
Experienced a previous opioid overdose, documented or self report	32 (43)
Reported daily milligrams* of unregulated fentanyl (Mean ± SD)	988 ± 1474
Smoking	48 (64)
Intravenous	26 (35)
Intramuscular	2 (3)
Insufflation	7 (9)
Oral	2 (3)
Reported daily milligrams of safe supply hydromorphone (Mean ± SD)	30.4 ± 45.8 (*n* = 5)
Active Medication for Opioid Use Disorder (MOUD) prescription at time of admission – Any dose	32 (43)
Methadone	25 (33)
Sustained release oral morphine	3 (4)
Fentanyl Patch	4 (5)
Historical trials of MOUD, including active prescriptions - Any dose	66 (88)
Buprenorphine/naloxone sublingual (Bup-SL)	35 (47)
Bup extended-release depot (Bup-XR)	7 (9)
Methadone	49 (65)
Sustained release oral morphine	20 (27)
Intravenous hydromorphone MOUD (iOAT)	1 (4)
Fentanyl Patch	4 (5)
Other routine substance use at time of admission	
Alcohol	15 (20)
Cannabis	11 (15)
Stimulant	60 (80)
Nicotine	37 (49)
Benzodiazepine/Sedative	14 (19)
**Past Psychiatric History**	
Mood Disorder	25 (33)
Anxiety Disorder	15 (20)
Psychotic Disorder, including substance-induced	29 (39)
Trauma-related Disorder	14 (19)
**Reasons for Admission (multiple may apply per patient)**	
Acute Kidney Injury	2
Bleeding	2
Bowel Perforation	1
Burns	1
Decreased level of consciousness (except overdose)	2
Heart Failure	1
Infection/Sepsis/Pneumonia/UTI/Osteomyelitis/Endocarditis	15
Leg Swelling	1
Liver Disease	2
Mental Health/Psychosis	21
HIV Neuropathy	1
Overdose	6
Pain	7
Rectal Prolapse	1
Seizure	3
Spine Spondylosis	1
Substance Intoxication	4
Substance Withdrawal	7
Trauma	4

### Completion of buprenorphine initiation

In total, 55/75 (73%) of patients were successfully initiated and discharged on Bup MOUD (Bup-XR *n* = 43, SL-Bup *n* = 12). The most common patient-reported reasons for unsuccessful initiation were preference to switch to another MOUD (*n* = 11) and withdrawal (*n* = 6). The remainder of reported reasons are outlined in Table 2. Seven patients self-directed their discharges before initiations were completed. There was no significant relationship between the three different protocols and rates of successful initiation (Fisher’s exact *p* = 0.191).

**Table 2:** IPPAS TD-Bup protocol outcomes

Outcomes	No. (%)
Successfully initiated onto Bup MOUD	55 (75)
SL-Bup initiation	12
Bup-XR initiation	43
Reasons for unsuccessful initiation onto Bup MOUD*	
Self-initiated discharge with any documented withdrawals	3
Self-initiated discharge without withdrawals	4
Withdrawals	6
Preferred to switch to another MOUD	11
Declined MOUD	1
Painful injection	1
Inferior pain control compared to methadone	1
Aborted for medical instability	1
Pruritis from patches	1
Self-initiated discharges after successful initiations	10
Self-initiated discharge with any documented withdrawals	4
Self-initiated discharge without withdrawals	6
All-cause COWS score increase > 6, or flagged for withdrawal symptoms on narrative charting review (Additional Table 1)	19 (25%)
Possible precipitated withdrawal	3
Unmet opioid deficit^**+**^	8
Opioid withdrawals occurred outside of Bup initiation protocol period	3
COWS elevation driven by causes other than opioid withdrawals	4
Not enough information	1
Insufficient COWS scores with no contributory charting notes	3

*Patients could report more than one reason

^**+**^ evidence of markedly reduced opiate intake with corresponding symptoms not correlated to Bup exposure

### Observed withdrawal symptoms

28 patients were found to have an adequate number of COWS scores for analysis (at least one between each scheduled intervention point). 25 patients had some but an inadequate number of COWS scores, and 22 patients had no COWS scores documented at all.

**Fig. 3:**
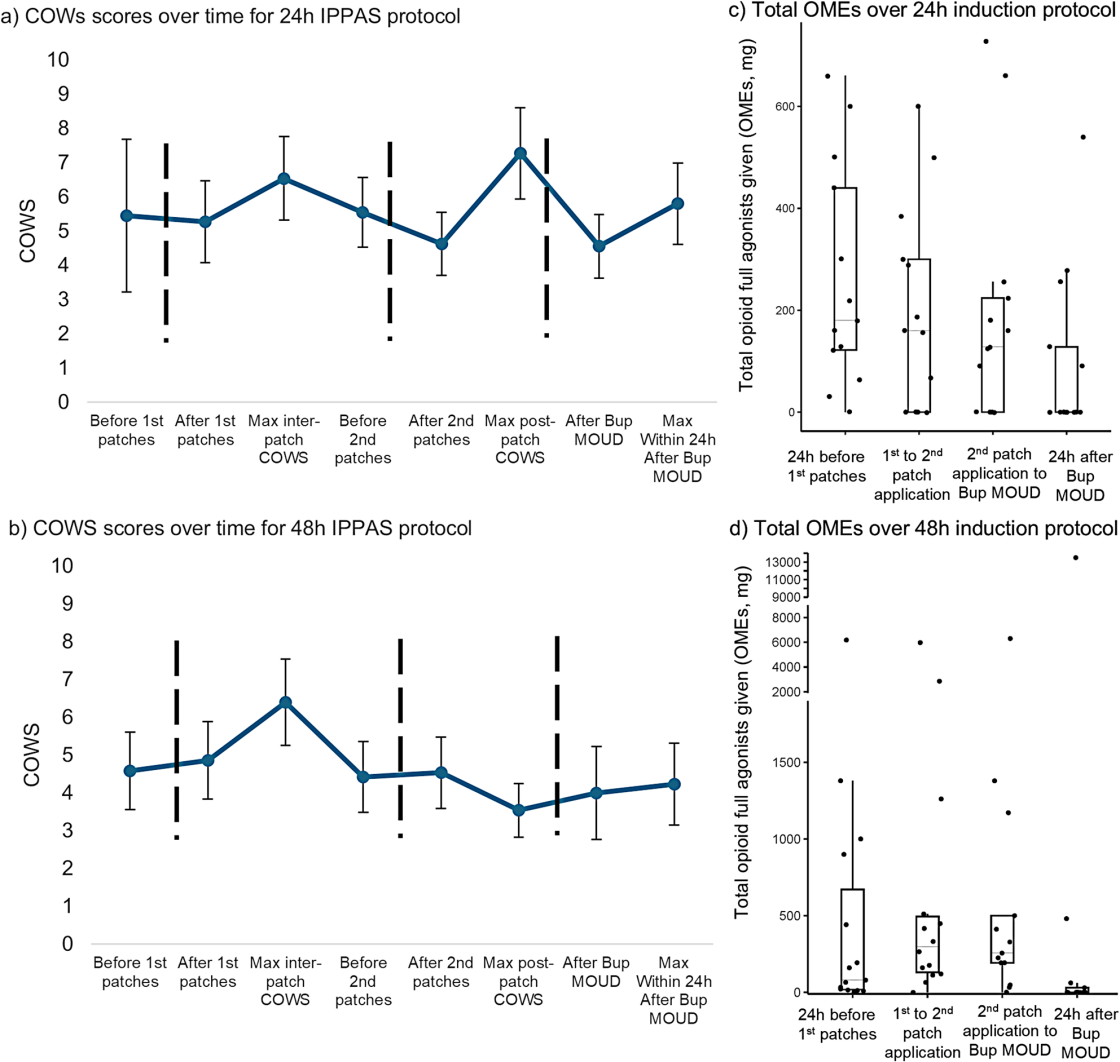
COWS scores and full agonist opioid use in 24 h (*n* = 13) and 48 h (*n* = 15) IPPAS protocols. The 24 h protocol (**a**) Separates patch applications by 12 h, and the 48 h protocol (**b**) Separates patch applications by 24 h. Dashed vertical lines indicate scheduled protocol intervention steps. Clinical opiate withdrawal scale (COWS) scores shown as means ± SEM. Accompanying box and whisker plots for median oral morphine equivalents (OMEs) use per indicated time period for the 24 h hour protocol (**c**) and the 48 h protocols (**d**). *n* = 13 for 24 h protocol (ac) and *n* = 15 for 48 h protocol (bd)

Among the 28 patients with adequate COWS scores, in both 24 h and 48 h protocols, mean COWS scores across time fluctuated between an average of 3.5 to 7.5 (Fig. 3 a,b). Seven cases of the 28 patients had COWS score elevations greater than 6 (3/13 patients administered the 24 h twice-dosed protocol and 4/15 administered the 48 h protocol. None received the 24 h thrice-dosed protocol); upon full review none were found to meet PW definition. In most cases, withdrawal was likely the result of inadequate opioid PRNs (Supplemental Table S1 for vignettes and rationales). The maximum COWS scores reached by each patient at any point in the protocol window (protocol itself, and 24 h pre- and post-protocol) ranged from 10 to 15.

Of the 25 patients with some but inadequate COWS scores, the maximum documented COWS score per patient at any time during initiation protocol including the periods 24 h before and after initiation was a mean (SD) of 6.2 (4.5), range 0–16. Two patients had a COWS score increase > 6 between any points of the protocol. These 25 were reviewed with full chart review along with the 22 patients with no COWS scores. Of the 47 patients, 3 lacked any contributory notes or data, 31 were found to have clear documentation indicating no bothersome withdrawal symptoms, and 13 were documented to have any withdrawal-type symptoms, including the two patients with withdrawals by COWS scores.

In total, within the limits of missing data, 19/75 (25%) patients either had a COWS elevation > 6, or were identified on chart review to have experienced any opioid withdrawals (Table 2), with 3 patients representing possible to probable precipitated withdrawal on clinician consensus review. Two of these experienced PW rapidly after receiving their 300 mg Bup-XL injections, and one experienced PW shortly after first application of patches. All three cases had confounding factors, such as delirium or pain, that resulted in uncertainty (Supplemental Table S1). There was no significant difference between protocols for proportions of all-cause withdrawals (Fisher’s exact *p* = 0.621) or possible precipitated withdrawals (*p* = 0.440). There was a trend for an association of all-cause withdrawals with rates of unsuccessful initiation (*p* = 0.082), and a significant association of all-cause withdrawals with self-directed discharges (*p* = 0.042). Of the 17 patients with self-directed discharges, 10 were successfully initiated on Bup OAT (4 with withdrawals, 6 without), and 7 were not successfully initiated on Bup OAT (3 with withdrawals, 4 without). On review, none of these patients had possible PW. In comparison, 12/58 patients without self-directed discharges experienced all-cause withdrawals.

### Opioid continuation with full agonists during buprenorphine initiation

Almost all patients received oral hydromorphone as opioid PRNs during the protocols. Four patients required prescribed IV fentanyl (average dose ranges of 44–477 µg/h IV) on admission for very high opioid tolerances. 26 patients were continued on pre-existing methadone prescriptions during the initiations (dose ranges 20 mg-160 mg daily; median (IQR) = 50 (40, 70) mg), and 16/26 patients (62%; Fisher’s exact comparison to those not taking methadone, *p* = 0.107) successfully initiated Bup MOUD. 3 of 3 patients were successfully rotated from SROM (500 mg, 950 mg, and 1000 mg daily) to Bup-XR. 4 of 4 patients using fentanyl patches (25 µg/h 75 µg/h, 500 µg/h, 900 µg/h) were successfully rotated to Bup-XR.

Plots of total prescribed OMEs (scheduled plus PRN) over time are shown in correspondence to COWS scores over time (Fig. 3 c,d). For the 48 h protocol (3d), there was a significant effect of time on opioid dose (F_3,33_ = 3.288, *p* < 0.05) after excluding one patient who received very high dose prescribed IV fentanyl (13500 mg OMEs) as an outlier. There was similarly a significant decrease in opioid use over time for the 24 h protocol (Fig. 3c, F_3,36_ = 3.535, *p* < 0.05). Of note, we captured some patients that received full agonists beyond the initiation of Bup MOUD for a few hours before orders were changed to only administrate Bup PRNs with discontinuation of all full agonists.

### Clinical adherence to TD-Bup protocol

16 patients met definition for patch timing deviations (>3 h late or early), and 32 patients met definition for Bup MOUD timing deviations (>1 h late or early). Fisher’s exact test showed no association between rates of all-cause withdrawal and whether there were protocol deviations for patch timing (*p* = 0.558) or Bup MOUD administration timing (*p* = 0.571). There was, however, a significant association of unsuccessful initiations with patch timing deviations (*p* = 0.008) and Bup MOUD timing deviations (*p* < 0.001). In patients without patch timing deviations, successful initiation rate was 48/59 (81%), versus 7/16 (44%) for patients with patch timing deviations. In patients with no Bup MOUD timing deviations, successful initiation rate was 35/37 (94%), versus 15/32 (46%) with timing deviations. 6 patients had no timing data available for the start of Bup MOUD, of which 5 were successfully initiated.

In six cases, single SL-Bup doses were given during patch initiation instead of after Bup MOUD was started, representing an additional type of protocol deviation. In two cases, 1-2 mg SL-Bup was given because the patients had lost one of the 12 patches. This did not cause any withdrawals. In the third case, Patient A in Additional Table S1 experienced elevation in COWS scores before second patch application, then received 2 mg SL-Bup erroneously with a corresponding decrease in COWS score. In another two cases, after uneventful patch initiations, all patches were removed prematurely before Bup-XR arrived on unit, and SL-Bup (2 mg) was used to bridge until the injection was given. The sixth patient had an incorrect order entry such that 8 mg SL-Bup was given before, instead of after, the patch protocol; the patient did not experience any withdrawals.

### Adverse events

Adverse effects are shown in Additional Table S2. Of note, we were not able to differentiate symptoms experienced related to other medical causes and included any reports of symptoms that were present. Adverse events were documented in association with 5 dropouts from initiation, with reports of agitation (*n* = 4), increased pain (*n* = 4), insomnia (*n* = 2) and pruritis (*n* = 1) as the reported causes (individuals could report multiple adverse events). No patients required naloxone resuscitation.

## Discussion

These IPPAS protocols are a means to convert the discreet steps of a low-dose Bup initiation protocol into a smooth integral over time. As there are no pauses to drug delivery and only 2 or 3 “boluses” with patch applications, this offers a potentially attractive way to reach optimal drug levels over a short period of time, limiting the exposure to rapid rises in serum levels and thus decreasing the risk for PW. A faster protocol also reduces the time a patient must wait to stabilize onto Bup MOUD, which can help reduce the risk of dropout and relapse. The findings of this retrospective chart review show that most patients who were treated with the TD-Bup initiation protocols successfully initiated Bup MOUD with a low incidence of withdrawal symptoms.

Employing adequate doses of short-acting full-opioid agonist medication throughout the initiation process constitutes a critical aspect of this protocol. Patients using daily fentanyl usually have high opioid requirements and can go into withdrawal within hours of last use. It is imperative to meet opioid requirements with full opioid agonists when serum Bup levels are still too low for the first hours. In our experience, when patients received inadequate full opioid agonist PRNs relative to their anticipated opioid requirements, they experienced withdrawal that was readily reversed by a few doses of full agonist PRNs. For this reason, we used a more specific definition of PW in this study by adding two criteria: it should not occur in the context of opioid deficit, and/or it needs to be resistant to rescue by full agonists. Capturing this difference is important since it has significant implications for management. Applying our definition of PW, we found that only 3/75 patients had possible or probable PW, with no unequivocal cases of PW. A key limitation is that determining whether withdrawal is precipitated can be nuanced and open to subjectivity. We have provided patient vignettes in Table S1 for interested readers to examine further for this reason.

Even if not precipitated, all-cause withdrawals, which occurred 25% of the time, were still significantly associated with negative outcomes. Roughly 41% (7/17) of the patients with self-directed discharge had withdrawals, compared to 21% (11/58) of patients who did not self-direct discharge, although there is no way to compare intensity of withdrawals due to missing COWS scores. In many of these cases, patients did not receive adequate or any opioid PRNs for withdrawal management and left hospital before this could be rectified, illustrating the concept that parallel administration of full agonists is probably necessary. Unmet opioid requirements is a major driver for self-directing discharge which occurs in 19–43% of admissions for this population [25, 26]. The observation that roughly three quarters of the cohort experienced no withdrawals and did not self-direct discharge, suggests that these protocols are likely overall beneficial.

The patients in this study seem to have more severe OUD and high prevalence of other social determinants of health relative to the available provincial statistics, which do not differentiate inpatient and outpatient populations. For instance, among people with OUD who overdosed on opioids in 2017 in BC, 30% reported unstable housing [27], compared to about 70% in this cohort of only inpatients. The provincial rate of concurrent stimulant use among patients with opioid use was 37% [28], whereas in this study it was 80%. As of 2022, less than 60% of all MOUD treatment courses in BC completed initiation and only half of successful initiations reached a minimum effective dosage. [29] The study patients primarily had severe OUD and very high opioid tolerance, as well as known factors associated with poorer treatment outcomes and retention [27, 28]. Despite poorer prognostic demographic factors, successful initiation rates (~73%) with these protocols are comparable or superior to provincial rates, albeit comparing across different patient settings.

The TD-Bup protocols streamline the initiation process compared to previously introduced low-dose initiation methods [10], dramatically reducing the number of doses and chances for administration errors that could lead to withdrawal and drop-out. While TD-Bup patches are expensive, they can shorten admissions and reduce system costs if patients are at high risk for being lost to follow-up and otherwise need longer inpatient stays to stabilize on MOUD. Moreover, it minimizes sleep disruption caused by initiation strategies that dose frequently, such as every 3 hours^26^ and greatly reduces the nursing workload. The large number of patches was well-tolerated and we did not experience any issues with patient discomfort or diversion. There was nevertheless an association of timing deviations with unsuccessful initiation onto Bup MOUD, perhaps as a predictor of patient ambivalence towards OUD treatment, or loss of confidence in the experimental protocol if treatment was not delivered as counseled. This finding suggests that it is of genuine importance to adhere to protocol timing during the initiation process.

Some limitations of our study is that it is an uncontrolled observational study in a special population with very high disease burden, so the usefulness of the protocols in a broader context remains to be seen. Furthermore, roughly two-thirds of our study patients had missing documented COWS scores that necessitated subjective clinician review, which was a possible source of bias. During the study period, we transitioned from paper to an electronic health record system, which may have introduced barriers to easily document COWS scores despite corresponding orders, as almost all paper records, in contrast to the electronic system, had complete sets of COWS scores. There may also be a temporal relationship, with comfort with the protocol and perceived low risk of withdrawal leading to less rigorous documentation among non-study clinical staff. In the absence of COWS scores over time, the binary presence or absence of withdrawals becomes susceptible to confounders. For example, it is possible that more-engaged patients were more likely to complete initiation and to make their withdrawals known to providers, whereas less-engaged patients may be less likely to make their withdrawals known and more likely to self-direct discharge. Retention on Bup MOUD beyond the first doses is important for patients, but we were not able to access patient information after discharge to report this, and many of these patients were started on Bup MOUD with the protocols just before discharge. Length of retention after initiation would be an important outcome for future studies with these protocols.

## Conclusion

The IPPAS TD-Bup initiation protocols led to 73% of patients successfully transitioning to Bup MOUD within 24 h or 48 h. While precipitated withdrawals were rare, all-cause withdrawals occurred in 25% of patients largely from unmet opioid requirements and was associated with self-directed discharges. There were no major adverse events. Further prospective studies are urgently needed to fully characterize the safety, efficacy and cost-effectiveness of this approach.

## Electronic supplementary material

Below is the link to the electronic supplementary material.

Supplementary material 1

Supplementary material 2

Supplementary material 3

## Data Availability

In accordance with research ethics requirements, the data from this study are not available to individuals outside the research team approved by the University of British Columbia Clinical Research Ethics Board.
