# Supplementary material for: 24-48 h initiation by transdermal buprenorphine for the treatment of opioid use disorder in the inpatient setting: a retrospective chart review
Source: Addict Sci Clin Pract. 2026 Mar 13;21:30. doi: 10.1186/s13722-026-00657-3 (PMC13020101; doi:10.1186/s13722-026-00657-3)
Supplement: Supplementary file 1 — Supplementary material 1 [file 13722_2026_657_MOESM1_ESM.pdf]

Supplemental Figure S1. Individual traces of COWS scores over time for each patient.

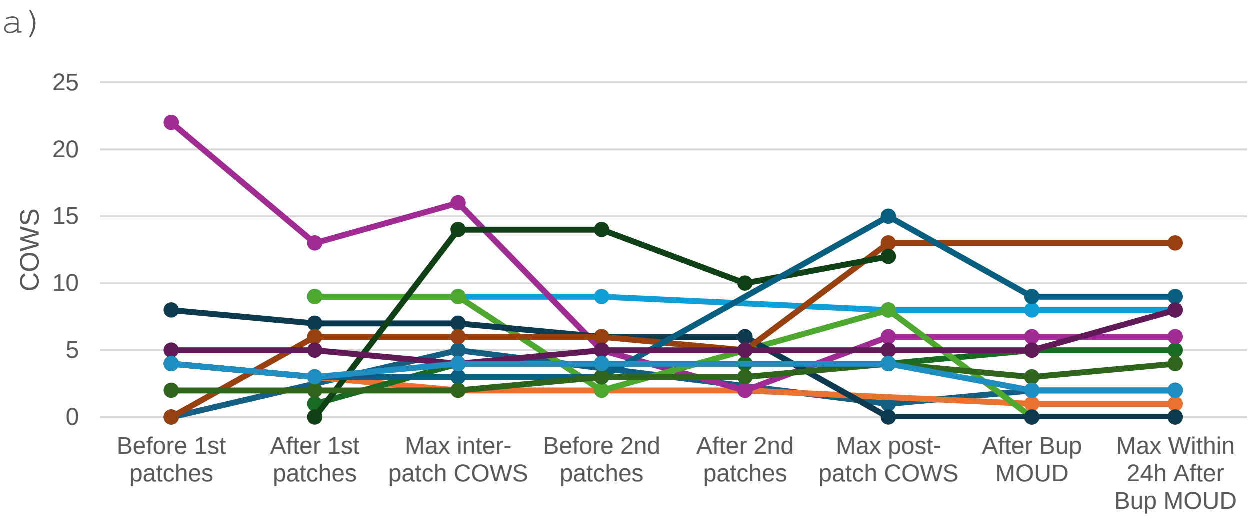

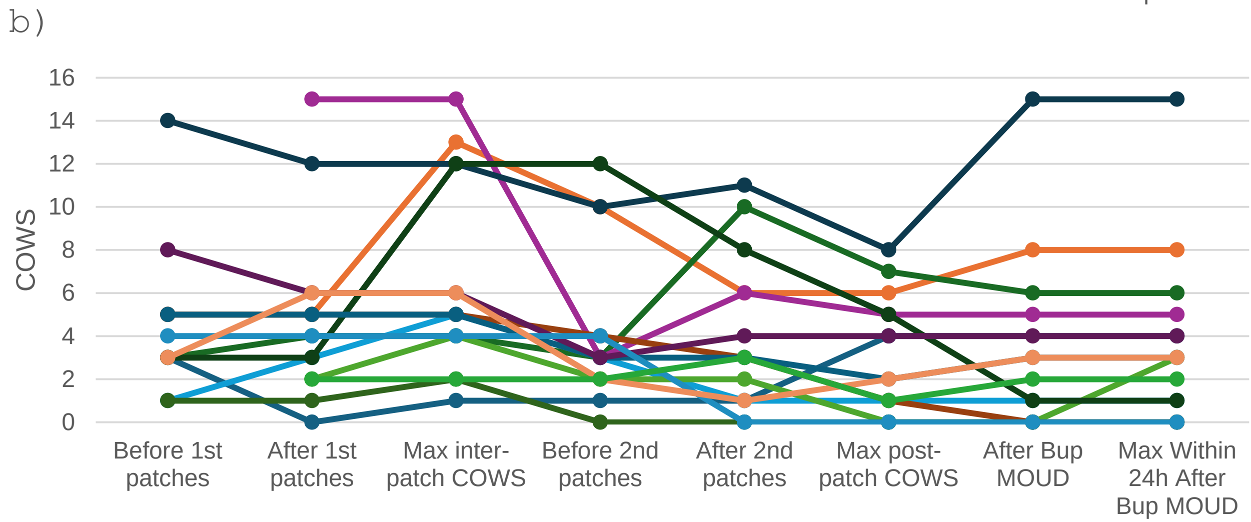

a. Individual traces for COWS scores over time for patients in the 24h induction protocol. b. Individual traces for COWS scores over time for patients in the 48h induction protocol. In both line graphs, each color represents an individual patient. Missing COWS scores are indicated by absence of small circles and connecting lines.
