# Supplementary material for: 24-48 h initiation by transdermal buprenorphine for the treatment of opioid use disorder in the inpatient setting: a retrospective chart review
Source: Addict Sci Clin Pract. 2026 Mar 13;21:30. doi: 10.1186/s13722-026-00657-3 (PMC13020101; doi:10.1186/s13722-026-00657-3)
Supplement: Supplementary file 2 — Supplementary material 2 [file 13722_2026_657_MOESM2_ESM.docx]

Table S1: Case descriptions for patients reviewed by clinician consensus where there was

indication for any withdrawals, or there was a COWS score of 6 or higher.

| Patient | Protocol | Case Description |
| --- | --- | --- |
| A | 48h | 39-year-old Aboriginal female, unstably housed, with severe OUD admitted for pneumonia. She was not on any MOUD and was smoking and injecting unregulated fentanyl daily. In the 24h pre- initiation, she received hydromorphone 6mg oral and 4mg IV for withdrawal. During initiation, her COWS scores were 5 both one hour before and after the first set of patches but rose from 5 to 13 several hours later in between the first and second set of patches before dropping to 11 one hour before the second patch application. She received zero PRNs during this period between the first and second patches. After the second set of patches were applied and she received 8mg oral hydromorphone and 2mg SL-Bup, and her COWS dropped to 6-8 through the rest of the initiation. She received 300mg of Bup depot and COWS score was a maximum of  8 after injection. |
|  |  | Conclusion: Inadequate hydromorphone between first and second patch application resulted in opioid deficit withdrawal. Timing does not suggest precipitated withdrawal. Protocol deviation involving premature administration of SL-Bup PRN occurred, but with no worsening of COWS score.  Max COWS score reached = 13 |
| B | 48h | 28-year-old male, unstably housed, with moderate OUD admitted for psychosis. He was smoking and injecting fentanyl and stimulants and not prescribed MOUD. He last used unregulated fentanyl about 13 hours before initiation start alongside a total of 2mg oral hydromorphone given in hospital, and his pre-initiation COWS scores was 3. Between first and second patch application, he received a further 64mg oral hydromorphone and had a maximal COWS of 4 during this period. After the second set of patches, his COWS rises from 3 to 10, the largest increase over his entire initiation. He received a further 120mg oral hydromorphone and his COWS score dropped to 7. He was initially started on 8mg BID sublingual Bup after initiation, and COWS score was a maximum of 6 in the 24h after successful initiation. He was then started on 300mg Bup depot after two days and sublingual Bup was  discontinued. |
|  |  | Conclusion: This patient has high opioid tolerance and requirements, but he experienced little withdrawal and opioid requirements at the beginning of initiation likely due to recent use of fentanyl. Inadequate hydromorphone between second patch application and injection (37-51 hours post-fentanyl) resulted in opioid deficit withdrawal as more time passed from when he last used large amounts of unregulated fentanyl. His highest COWS  score was 10, which is still mild and not very negatively impactful. |

|  |  | Max COWS score reached = 10 |
| --- | --- | --- |
| C | 48h | 37-year-old Caucasian female, unstably housed, with moderate OUD admitted for unspecified psychosis. She reported smoking and injecting 1g of unregulated fentanyl daily and did not use MOUD. Her UDS was positive for amphetamine, cocaine, cannabis, fentanyl, opioids, and benzodiazepines. During the 24h before initiation, she received a total of 20mg of hydromorphone orally as PRNs, but her COWS score remained elevated at 14 before initiation start. After first patch application, her COWS score was between 10-12, and she received 104mg of oral hydromorphone during this period. COWs decreased to 8 after second patch application accompanied by another 48mg of oral hydromorphone. After 300mg Bup-XR was injected, within 10 minutes her COWS score rose from 8 to 15. Her COWS 12h later  had dropped to 6 without any further opioid PRNs. |
|  |  | Conclusions: This patient began initiation in moderate withdrawal that improved over time. Her increase in COWS score with injection was most likely a pain reaction. Her withdrawal exacerbation after injection of Bup depot occurred in less than 10 minutes, and resolved quickly without any PRNs, which is not likely to be induced by the depot, according to prior pharmacokinetic modeling.  Max COWS score reached = 15 |
| D | 48h | 42-year-old female with unstable housing and high dose fentanyl use not on MOUD admitted for infectious colitis. Her COWS scores were 3 before and after first patch application. She received 3mg of hydromorphone in the 24h before initiation, as well as 4mg after first application but no other full opioid agonists. Before second patch application, her COWS climbed to 12. She was started on prescribed IV fentanyl ^26^, receiving 350µg/h for 3 hours, second application of patches was applied, and she received 300µg/h until injection of 300mg of Bup depot. COWS scores were 8 after fentanyl infusion and the second patch application, and 5 before Bup-XR 300mginjection. Post-injection, fentanyl infusion was stopped, and COWS score was 1, and next morning her  COWS was 0. |
|  |  | Conclusion: High opioid requirements that went unmet about 9-10 hours into the protocol with inadequate hydromorphone PRNs administered. More consistent with opioid deficit withdrawal than precipitated withdrawal caused by patches based on timing.  Maximum COWS score reached = 12. |
| E | 24h (q12h) | 56-year-old male, unstably housed, with severe OUD, smoking 0.5g of unregulated fentanyl daily, admitted for substance intoxication. He was taking 40mg of methadone daily. 24h prior to initiation, he received 80mg of oral hydromorphone plus 40mg of  methadone for withdrawal management. Methadone was |

|  |  | intentionally not discontinued during initiation. After first application of patches, he received a further 24mg of oral hydromorphone and 20mg of methadone. After second application of patches, he received 16mg of oral hydromorphone and another 20mg of methadone. COWS scores were between 3-6 the entire initiation up until the point of Bup injection. However, he changed his mind and wished to return back to methadone, and then experienced withdrawals, with a COWS score increase from 5 to  13 when Bup patches were discontinued. |
| --- | --- | --- |
|  |  | Conclusion: Withdrawals were associated discontinuation of Bup patches and do not represent precipitated withdrawals.  Maximum COWS score reached = 13 |
| F | 24h (q12h) | 57-year-old male admitted for suicidal ideation, who has a severe opioid use disorder smoking about 0.25g of unregulated fentanyl daily. He was taking 100mg of methadone daily and wished to rotate to Bup sublingual. His COWS scores before and after first patch application were 0. Between the first and second patch applications, his COWS score climbed to 14. He had received no opioids except his scheduled 100mg of methadone daily up until this point. He received a total of 56mg of oral hydromorphone between second patch application and Bup depot, which decreased his COWS scores to 10. He was then offered 8mg of suboxone but refused it and then left the hospital. Total length of stay was 1 day. |
|  |  | Conclusion: Significant opioid requirements that were not met with full agonist PRNs after admission to replace an equivalent of 0.25g of unregulated fentanyl. The time course is also more consistent with opioid deficit withdrawal than precipitated withdrawal, as there were no clear peaks in COWs scores within 1-2 hours after each patch application.  Maximum COWS score reached = 14 |
| G | 24h (q12h) | 33-year-old male with unstable housing and admitted for foot osteomyelitis. He has a severe OUD and smokes 1g of unregulated fentanyl a day and takes a prescribed 40mg of methadone a day. In the 24h before initiation, he received a total of 16mg of subcutaneous hydromorphone. He did not receive any methadone. Pre- and post-application of the first set of patches, his COWS scores were 4 and 3 respectively. He receives a further 16mg of subcutaneous in the interval between second patch application and Bup-XR injection. Close to the time of patch removal and Bup depot injection, the patient then had an escalation of COWS from 3 to 15, which dropped to 9 in 1 hour, before declining to 0 within the span of 5 hours. The patient was not successfully induced onto Bup, as he chose to return to 40mg methadone. |
|  |  | Conclusion: Received very little full agonist (total of 16mg subcutaneous hydromorphone during initiation with no  methadone), despite heavy fentanyl use and a methadone |

|  |  | prescription before admission. Likely opioid deficit withdrawal that improved after  Maximum COWS score reached = 15 |
| --- | --- | --- |
| H | 24h (q12h) | 24-year-old female with unstable housing admitted for precipitated opioid withdrawal from a few days before. She used 1g/day of IV unregulated fentanyl with the last dose 48 hours before presentation. After precipitation, she had been started on methadone 50mg with last dosage 3 hours before initiation. In the 24h before TD-Bup initiation, she received a total of 40mg of oral hydromorphone, 104mg of subcutaneous hydromorphone and 110mg of methadone, with the most recent PRN opioid prior to initiation being 0.33hrs. Between the first and second set of patches she received 48mg of oral hydromorphone, between the second set of patches and the administration of Bup-XR 300mg she received 16mg of oral hydromorphone. Her COWS score was 5 before the first set of patches and increased to 11 after the first set of patches, but she reported feeling positive towards the treatment and reported no symptoms after second set of patches. She was successfully transitioned and discharged on Bup-XR. The total time from first set of TD-Bup initiation to administration of Bup-XR was 24.65  hrs. |
|  |  | Conclusion: Maximal COWS score of 11 with a decrease in opiates after patch initiation protocol began. Resolved with little additional intervention (16mg of hydromorphone), thus unlikely to represent precipitated withdrawal. |
| I | 48h | 42-year-old aboriginal male with unstable housing admitted for infective endocarditis, using 0.3g/day of unregulated fentanyl per day via smoking and IV, last dose unknown. He was on methadone 75mg PO daily before initiation. In the 24 hours before TD-Bup initiation to first patches, he received a total of 12mg of oral hydromorphone and 75mg of methadone, with the most recent PRN opioid prior to TD-Bup initiation being 4.5hrs. Between the first to second set of patches, he received 20mg of oral hydromorphone, but then had all full opioid agonist orders erroneously discontinued. He had a COWs score of 1 and 0 before and after the first set of patches and rose to a maximum score of 15 9 hours after the first set of patches. The patient received only 1 set of patches and then left AMA due to withdrawal and pain. |
|  |  | Conclusion: Full agonists were incorrectly discontinued in the first 24h of the protocol, leaving him at an opioid deficit. COWS score Peaked 9 hours after the first set of patches after his methadone was erroneously discontinued. His 20mg of hydromorphone over 9 hours is not a significant dose compared to his baseline of 75mg of methadone daily. Withdrawals also occurred well beyond the expected timepoint for precipitated withdrawal. This was opiate  deficit withdrawal. |

| J | 48h | 71-year-old male with unstable housing admitted for unspecified psychosis, stimulant use disorder, and OUD. He was also agitated and delirious, with pain is his left leg. He used unregulated IV fentanyl of an unknown amount daily. He was taking methadone 135mg daily, time of last dosage unknown. He was not taking methadone in hospital. No COWS scores available for this admission.  He received 24mg oral hydromorphone between the first and second set of patches, 144mg between the second set of patches to administration of SL-Bup, and 6mg between patch removal to 24 hours after removal.  Approximately 30 mins after the first set of patches, he endorsed severely worsened pain and discomfort related to withdrawals. He received 24mg of oral hydromorphone in the 24h between first and second set of patches. He also used illicit opioids during initiation. The next available notes are 6h later, indicating ongoing agitation, though also notes that he was delirious. By 24h later, he had received the second set of patches and continued to report intolerable leg pain and request pain medications. Between the second set of patches to the end of the protocol, he received and tolerated another 144mg of oral hydromorphone in 24h. He was first started on SL-Bup MOUD, before transition to and discharge on 300mg Bup-XR. The time from the first set of TD-Bup patches  to 300 mg of Bup-XR was 69.3 hours. |
| --- | --- | --- |
|  | (PW) | Conclusion: This case had possible precipitated withdrawal. Without COWS scores and a breakdown, it is difficult to determine whether symptoms were from confounding delirium, psychosis, leg pain, or opioid withdrawals. The symptoms do correlate with the expected time course for precipitated withdrawals (30 mins after first patches). He was also significantly under-dosed with full agonist opioids with 24mg in the first 24h, given he tolerated 144mg of hydromorphone the next day, as an alternative explanation. |
| K | 48h | 47-year-old Aboriginal male with unstable housing admitted for abdominal pain. He had severe OUD with unregulated smoked fentanyl at 0.3g/day. He had been on MOUD before, including buprenorphine/naloxone 32mg and methadone 160mg. 24h before the first set of patches, his COWS score was 6 and he received 22mg of oral hydromorphone and 10mg of methadone, with the most recent PRN opioid before initiation being 1.5 hours. His COWS rose to 7 from 6 after the first set of patches. There are no COWS scores available after that. Between the second set of patches to administration of Bup-XR, he received 16mg of oral  hydromorphone, and from patch removal to 24 hours after patch |

|  |  | removal, he received 8mg of oral hydromorphone. Mild withdrawal symptoms with no POW were documented narratively. He was successfully transitioned to 300mg Bup-XR after 49hours from start of initiation and was discharged on 300mg Bup-XR.  He experienced some moderate sedation 6 hours before administration of Bup-XR, not requiring naloxone or resuscitation. |
| --- | --- | --- |
|  |  | Conclusion:  No evidence of significant withdrawals on narrative review. |
| L | 48h | 25-year-old white male with unstable housing, admitted for abdominal pain thought possibly gastroenteritis. He had severe OUD. He used unregulated smoked fentanyl 2g/day. He was not on MOUD but had prior trials of SL-Bup 24mg and methadone 110mg. 24 hours before administration of first set of patches, his COWS was 4 and he received 15mg of oral hydromorphone, with no unregulated opioids used before initiation. No other COWS scores available.  One day after the patches were started, he was found using substances in the hospital washroom, then was noted to be acutely psychotic, with delusions about a “crypto scam”. Psychiatry was consulted and certified him under the mental health act. He was nonetheless continued on the initiation protocol, albeit with significant delays. He received 15mg oral hydromorphone between the first to second set of patches (second set applied at 44h, thus 20 hours late) and 75mg between the second set of patches to administration of 8mg SL-Bup (given at 76h, thus 8h late).  There were subjective withdrawals noted without objective signs documented, and no signs of autonomic elevation in his vital signs. He also was experiencing diarrhea and flu-like symptoms in the context of gastroenteritis. He was successfully started on 8mg SL- Bup but apparently self-directed discharge before further titration. Time from first set of TD-Bup patches to SL-Bup administration was 76.9 hrs. He apparently tolerated the 8mg SL-Bup without any signs or symptoms of withdrawal. |
|  |  | Conclusion: This case had a significant protocol deviation due to emergence of probable stimulant-induced psychosis that significantly interrupted timing, and is not representative of any of the patch protocols described. He experienced withdrawals throughout the patch period and got very little full agonist opioids in the first 44h of the protocol. It’s likely this was opiate deficit  withdrawal since Bup levels did not rise as much as intended and his hydromorphone usage was quite low. |
| M | 48h | 34-year-old female with unstable housing, admitted for |

|  |  | sepsis/OUD/ Liver disease NOS/ Hematological disorder NOS. She had pre-existing pain in her knees and ankles. She used unregulated smoked and IV fentanyl 3g/day with last use 72 hours before initiation. She had a prior trial of methadone at an unknown dosage. In the 24h before TD-Bup initiation, she received a total of 76mg of oral hydromorphone, 0.4mg IV hydromorphone, and 6mg of subcutaneous hydromorphone, with most recent PRN opioid given 1.1h prior to initiation. It was noted that she was suspected to be using unregulated fentanyl on the wards periodically during the admission which may have continued through the initiation protocol. No COWS scores were available this admission.  Withdrawals were reported by the patient that started fairly quickly after application of the first set of patches and she was requesting high doses of hydromorphone PRNs. The symptoms were primarily pain of her ankles and knees, and cravings. She was screaming in pain and expressing a desire to leave. Our consult service reassessed and believed that the patient had very high opioid tolerance based on previous consultations and added PRN IV fentanyl to her regimen. She received 156mg oral hydromorphone, 10mg IV hydromorphone, 79mg of subcutaneous hydromorphone and 1200mcg of prescribed IV fentanyl in the time between first and second sets of patches.  She received 72mg oral hydromorphone, 3.5mg IV hydromorphone, 90mg of subcutaneous hydromorphone and 900mcg of prescribed IV fentanyl between the second set of patches to administration of Bup-XR. It took 43 hrs from administration of the first set of TD-Bup patches to injection of Bup-XR. She was successfully transitioned to 300mg Bup-XR but self-discharged afterwards.  Adverse Symptoms: Some periods of sedation not requiring naloxone resuscitation (felt secondary to sepsis by internal medicine), worsened pain, and agitation. |
| --- | --- | --- |
|  |  | Conclusion: This patient was an unwell (septic) patient with chronic pain, self-managing with high-dose unregulated fentanyl in the community. Review of her usage totals for prescribed opioids reveals that her tolerance was very high and not adequately met in hospital. Upon starting patches, she likely already had an opioid deficit presenting primarily as inadequate analgesia. She was prescribed IV fentanyl, which was effective given that her doses decreased over time.  The timing for the start of withdrawals is soon after patch |

|  |  | application, which raises suspicion for precipitation. But, there was a lack of any significant features of opioid withdrawals except for pain and cravings. Overall, it was felt that this represented a pain crisis with opiate deficit, not precipitated withdrawals |
| --- | --- | --- |
| N | 24h (q12h) | A 27-year-old female with unstable housing was admitted for sepsis and opioid use disorder. She had OUD of unspecified severity, using both IV injection and insufflation of approximately 0.2g of fentanyl daily. She was on 100mg of 12h-sustained release oral morphine daily as her MOUD, she had a prior trial of methadone at an unknown dose. In the 24 hours before the start of the initiation, she received a total of 136 mg of po hydromorphone, 24 mg of IV hydromorphone, and 200 mg of 12h sustained-release oral morphine. COWS scores were not documented this admission.  She reported subjective withdrawals starting roughly 7 hours after the first set of patches. No objective signs documented. PRNs used between first and second set of patches totaled 48mg of oral hydromorphone, 10mg of IV hydromorphone, and 200mcg of IV fentanyl. After the second set of patches, she received an additional 10mg of IV hydromorphone and 200 mcg of IV fentanyl. There were no objective signs of withdrawals after the second set of patches except for a transient hypertension with systolic 146mmHg. Ultimately, the transition to a full dose of SL-Bup was unsuccessful, as the patient self-initiated discharge in this period. |
|  |  | Conclusion: She never demonstrated significant withdrawals on chart review. She developed some subjective opiate withdrawals at 7 hours into the protocol, which is too late to be precipitated by TD-Bup. Full opioid agonist usage totals dropping significantly after the start of patches suggests she could have been underdosed  after the first set of patches, explaining the subjective withdrawals. These were likely mild, if not accompanied by any objective signs. |
| O | 24 h (q12h) | A 46-year-old woman with unstable housing was admitted for treatment of rectal prolapse. She had a severe opioid use disorder and smoked 0.2 g of fentanyl daily. She had a prior trial of SL-Bup as MOUD. She was not on methadone at the time of admission but was rapidly increased to 30mg TID of methadone in hospital for pain control. 24-hours before the first set of patches she received a total of 96 mg of PO Hydromorphone, 40 mg of IV Hydromorphone, and 90 mg of methadone. Only one COWS score was recorded, approximately six hours before the first set of patches, with a score of 4. Between the first and second sets of the patches, she was given an additional total of 64 mg PO Hydromorphone and 30 mg of methadone. After the second set of patches, she received 32 mg of PO Hydromorphone, 6 mg of SC Hydromorphone, and 30 mg of methadone. There were no  withdrawals during this portion of the initiation. She had received |

|  |  | zopiclone the night before and was drowsy the second day of the initiation. Following her Bup-XR, which was given while asleep, the patient awoke and experienced anxiety and panic instantly, with increased cravings, yawning, restlessness, anxiety, and fidgeting afterward that peaked 30 minutes after receiving the injection. She received a total of 4 mg of PRN SL-Bup and another 4 mg the  following day. |
| --- | --- | --- |
|  | PW | Conclusion: Being woken by a Bup-XR injection could plausibly result in some pain, anxiety, and agitation that may confound opioid withdrawal symptoms, but the escalation up to 30 mins with a clear opioid withdrawal syndrome suggest this is probably precipitated withdrawal. An alternative contributing factor is the possibility she developed some opiate deficit while sleeping most of the day, unable to request PRNs, but she did not have  documented withdrawals until after Bup-XR was given. |
| P | 24h (q12h) | A 50-year-old woman with unstable housing was admitted for cellulitis. She had a severe opioid use disorder and consumed 3.5g of unregulated fentanyl daily. The patient had previous trials of SL- Bup and Bup-XR but was not on MOUD at the time of admission. Within 24 hours prior to her first set of patches, she received a total of 40 mg of PO Hydromorphone. She received no prn opioids between the first and second set of patches but was documented as using some unregulated fentanyl instead. After the second set of patches, she was given 8 mg of PO Hydromorphone, followed by 2 mg of SL-Bup after transition to Bup-XR. The only COWS score captured was 9 1h after the Bup-XR injection. She had some nausea and vomiting on the first day of induction, though a  concurrent bowel obstruction was also suspected at that time. |
|  |  | Conclusion: She did not experience an obvious opioid withdrawal syndrome. If the nausea/vomiting was related to opioid withdrawal, it is most likely due to opioid deficit, if she was previously using  3.5g of unregulated fentanyl daily, then received no hydromorphone for the first 12h of the initiation protocol. |
| Q | 24h (q12h) | A 33-year-old Aboriginal woman with unstable housing was admitted for substance-induced psychosis. She had a severe opioid use disorder, mainly smoking 0.25g of unregulated fentanyl daily. She was previously on SL-Bup as MOUD, but the dosage and timing of the last dose were unknown. There were no opioids given in the 24-hours before the first set of patches. The second set of patches was administered more than three hours later than the scheduled time. Between the first and second set of patches, a total of 8 mg of PO Hydromorphone was given. Following the second set of patches, the patient had a COWS score of 4, which remained the same after Bup-SL MOUD started at 8mg daily. 2 mg of SL- Bup was then administered as PRN for withdrawals. The patient  was discharged on 12 mg of SL-Bup. Only two COWS scores were |

|  |  | available: 8 before patch induction and 4 after the second set of patches. Overall, she did not experience significant withdrawal symptoms during the initiation process. There was a possible illicit drug use during initiation according to nursing notes and progress notes. |
| --- | --- | --- |
|  |  | Conclusion: Uncomplicated patch initiation over 24h. Notably, very little hydromorphone PRN use, probably as her unregulated fentanyl was adequate. Transitioned to 8mg of Bup-SL for MOUD, which proved subtherapeutic for her; titrated by PRNs up to 12mg as guided by withdrawal symptoms. No precipitated withdrawals here, and no significant withdrawals of any cause during initiation  period. |
| R | 24h (q12h) | A 29-year-old female with stable housing was admitted for epilepsy. She had an active OUD, unspecified severity. She smoked around 1g of fentanyl and heroin daily. She was on 25 mg of daily methadone for MOUD and had a prior trial of Bup-XR. She received 25 mg of oral methadone 24 hours before the application of the first set of patches. Between the first and second sets of patches, she was given a total of 8 mg of PO Hydromorphone.  Following the second set of patches, she received a total of 28 mg of PO Hydromorphone, 1 mg of SC Hydromorphone, and 25 mg of methadone. After the removal of the patches and within 24 hours post-removal, she received another 48 mg of PO Hydromorphone, 2 mg of SL SL-Bup, and 25 mg of methadone.  COWS scores were not recorded. The patient experienced severe withdrawal symptoms, including shaking, screaming in pain, and repeatedly requesting more methadone and PRN opioids after Bup-  XR was started. |
|  | PW | Conclusion: The initiation phase with patches was uneventful, but she appeared to experience severe subjective withdrawals after the depot injection. Conversely, pain and agitation were the main driving symptoms without evidence of other objective signs. She was also using few opioid PRNs in the first half of the protocol relative to endorsed consumption of unregulated opioids, possibly leading to an accumulating opioid deficit. On balance, it is probable that this was precipitated withdrawal. |
| S | 24h (q12h) | A 50-year-old Indigenous woman with stable housing was admitted due to psychosis and had a severe opioid use disorder. She uses fentanyl, approximately 0.1g daily, though the route of administration was not documented. She had previously been on 50 mg of methadone but was no longer on MOUD at time of admission. Before applying the first buprenorphine patch, the patient showed clear signs of withdrawal, such as mild sweating,  cravings, fatigue, agitation, and increased pain. 24 hours prior to |

|  |  | the first set of buprenorphine patches, the patient received 96 mg of PO Hydromorphone and 40 mg of Methadone. After the first set of patches, she was given 32 mg of PO Hydromorphone and 40 mg of methadone. No COWS scores were available. The patient still experienced the same level of withdrawals after starting the first set of patches, then she refused the second set, stating she “Did not feel well on it” and decided to switch back to methadone. |
| --- | --- | --- |
|  |  | Conclusion: She was in opioid withdrawals but had received a large amount of full agonist prior to starting the initiation protocol. The patches did not help with her withdrawals, as expected given the low serum levels at that stage, and PRN usage per hour decreased slightly. There was no precipitated withdrawal. She was originally in opiate deficit withdrawals and remained in opiate deficit withdrawals before stopping early on in the protocol. |
| T | 24h (q12h) | A 47-year-old South Asian male with unstable housing was admitted due to chest wall pain and pneumonia. He had an active opioid use disorder, though the severity was unknown. The patient smoked approximately 1g of fentanyl and heroin daily. He had previously tried MOUD including SL-Bup, Bup-XR, but the dosages were unknown. He was on methadone 70-80mg in community before admission and was titrated to 130mg this admission. He was having difficulty going to the pharmacy daily, so he wanted to rotate to Bup-XR from methadone.  In the 24 hours before his first set of patches, the patient received a total of 60 mg of PO Hydromorphone and 130 mg of methadone. After the first set of patches, he was given 130 mg of methadone. Following the second set of patches, he received an additional 12 mg of PO Hydromorphone. His COWS scoring was documented only once, after Bup-XR administration, and was 9. No further PRN opioids were provided 24-hours after the end of initiation.  The patient was successfully discharged on Bup-XR. |
|  |  | Conclusion: Mild withdrawals based on COWS scoring only after Bup-XR injection that resolved without any interventions. Does not represent precipitated withdrawals. May be related to fully stopping his methadone 130mg at that time. |
| U | 48h | A 34-year-old Indigenous woman with stable housing, living in a shared house with a roommate, was recalled from extended leave under the Mental Health Act for missing her depot antipsychotic injection for a psychotic disorder. She had severe opioid use disorder and primarily smoked fentanyl, amount unknown. She was not on MOUD but had previously been prescribed 400 mg of 24h sustained release oral morphine (SROM) daily. |

|  |  | In the 24 hours preceding her first set of patches, the patient received a total of 200 mg of oral SROM, with the most recent dose administered 1.3 hours before the first set of patches. She then received an additional 200 mg of oral SROM between the first and second sets of patches, and another 200 mg between the second set and the removal of the patches. The only COWS score available was 8 between the first and second sets of patches. Notes indicated that the patient did not experience withdrawal during the initiation process. Before the second set of patches were applied, it was noted that there were no acute withdrawal concerns, though she reported some subjective withdrawal symptoms without major  objective evidence. She was transitioned to 300mg of Bup-XL. |
| --- | --- | --- |
|  | nil | Conclusion: Transient and mild opiate deficit withdrawal early into the protocol, with resolution after further opioids given. Not characteristic of precipitated withdrawal. |
| V | 24h (q8h) | A 47-year-old Caucasian male with unstable housing was admitted for polysubstance use disorder. He sought treatment in the hospital and had an active opioid use disorder of unknown severity. The patient primarily smoked and injected 0.15g of unregulated fentanyl daily. Before initiation, he was not on any MOUD.  The patient did not receive any opioids in the 24 hours prior to initiation. The primary goal of the protocol was to initiate him on Bup-XR. During the initiation period, he received 16 mg of oral hydromorphone (PO) between the first and second sets of patches and another 16 mg between the second and third sets of patches. No additional PRN opioids were administered during this time. No COWS scores were documented throughout the initiation, and there was no evidence of precipitated withdrawal noted in his chart. |
|  |  | Conclusion: No evidence of withdrawals. |
| W | 24h (q12h) | A 34-year-old female with unstable housing was admitted for unspecified psychosis. She had documented stimulant and opioid use disorders of unknown severity. The patient primarily used fentanyl and heroin, on average $30-worth of “heroin” daily, which she injects, snorts, and smokes. The estimated amount is 0.1-0.2g a day. She had prior MOUD trials of an unknown dose of SL-Bup, methadone 30 mg, and SROM 300 mg.  The only PRN opioid the patient received was 24 mg of oral hydromorphone (PO) 24 hours prior to initiation. Following the patches, the patient did not start treatment with Bup-XR or resume SL-Bup. A COWS score of 2 was recorded both prior to the administration of the first set of patches and one hour after.  The patient exhibited persistent agitation and aggressive behavior toward staff throughout her stay, even before the administration of |

|  |  | the patches. She was placed in a seclusion room due to her aggression. Documentation did not indicate any subjective or objective withdrawal symptoms during the initiation process. The patient was eventually transferred out of hospital to the psychiatric intensive care unit. |
| --- | --- | --- |
|  |  | Conclusion: No evidence of any withdrawals during the initiation process and tolerated the patches well. Based on opioid usage history, she was not likely to be at risk of precipitated withdrawal, but could not be sure due to unreliability from psychosis. Lost to  follow-up after transfer. |
| X | 24h (q12h) | A 24-year-old male, living with his family in a house, was recalled from extended leave of the Mental Health Act. He presented to the hospital with suicidal ideation and homicidal ideation (threatening his parents) in the context of substance intoxication. The patient had severe opioid use disorder (OUD), primarily smoking unregulated fentanyl, as well as stimulant use disorder. He had previously been on MOUD such as methadone, SL-Bup, and Bup- XR.  The patient received 2 mg of PRN oral hydromorphone 24 hours before initiation. He then received 4 mg of PO hydromorphone between the first and second sets of patches. His only COWS score available was 6 between 1^st^ to 2^nd^ sets of patches. The patient reported mild cravings and minimal withdrawal symptoms at the beginning of initiation. He was successfully transitioned to Bup- XR after the patches and was discharged without significant complications. |
|  |  | Conclusion: None to mild withdrawals during initiation. No precipitated withdrawals. |
| Y |  | A 42-year-old Indigenous male with unstable housing was admitted for osteomyelitis. He had OUD, primarily smoking and injecting 0.1g of unregulated fentanyl daily. The patient had trialed SL-Bup, methadone, and SROM before, although the doses and the last dates of prescriptions were unknown. No COWS scores were documented in his chart.  The patient did not receive any PRN opioids in the 24 hours prior to initiation or during the initiation process. He underwent the initiation protocol and did not have documented reports or observations of opioid withdrawal. The patient did not transition to Bup-XR after initiation and removed 11 out of 12 buprenorphine  patches on his own. All 11 patches were re-administered |

|  |  | simultaneously shortly after, but the patient refused Bup and opted for SROM. |
| --- | --- | --- |
|  |  | Conclusion: No evidence of withdrawals, but did not complete initiation as intended. |
| Z | 48h | A 28-year-old Indigenous male with unstable housing was admitted for a new right MCA ischemic stroke secondary to prosthetic valve endocarditis. The patient was scheduled for an emergency cerebral angiogram and thrombectomy. He remained hospitalized to ensure the urgent procedure could be completed, and his opioid withdrawal symptoms needed to be managed accordingly.  The patient had severe OUD, injecting 2-3g of fentanyl daily. He was on 85mg methadone daily prior to admission, and his dose was restarted during his stay, which initially caused drowsiness. The CPAS team was consulted, and a Bup initiation was ordered to rotate to a less sedating MOUD given his stroke.  Before the administration of buprenorphine patches, the patient received no opioid medications other than his scheduled methadone dose. He was given 95 mg of methadone in the 24 hours prior to induction, 95 mg between the first and second sets of patches, and an additional 95 mg between the second set and the removal of the patches.  The patient did not experience withdrawal symptoms, cravings, or signs of precipitated withdrawal during the induction process. He remained compliant with treatment throughout his admission,  successfully transitioning to a total a final dose of 20mg Bup-SL daily at discharge. |
|  |  | Conclusion: Flagged for clinician review due to missing notes. No evidence of withdrawals on clinician review. |
| AA | 24h (q12h) | A 57-year-old Indigenous male with unstable housing was admitted for cervical spondylosis. He had severe OUD and experienced chronic pain due to central cord syndrome. The patient smoked 0.1–0.2 g of unregulated fentanyl daily. He had previous trials of methadone 60mg and SL-Bup 3mg BID as MOUD but was not on MOUD at admission. No COWS scores were documented.  During admission, the patient received a total of 12 mg of oral hydromorphone in the 24 hours prior to the administration of the first set of buprenorphine patches. The patient refused to apply the second set of six patches and requested the removal of the first six patches, because of itchiness at the application site. He was notably agitated while the patches were in place and rejected pain medications that morning. He did not transition onto Bup MOUD.  There was no documentation that he experienced any withdrawal |

|  |  | symptoms. He self-initiated discharge and was lost to follow-up. |
| --- | --- | --- |
|  |  | Conclusions: No documented withdrawals. He stopped his initiation protocol presumably for itchiness, not due to precipitated withdrawals. |
