# Supplementary material for: 24-48 h initiation by transdermal buprenorphine for the treatment of opioid use disorder in the inpatient setting: a retrospective chart review
Source: Addict Sci Clin Pract. 2026 Mar 13;21:30. doi: 10.1186/s13722-026-00657-3 (PMC13020101; doi:10.1186/s13722-026-00657-3)
Supplement: Supplementary file 3 — Supplementary material 3 [file 13722_2026_657_MOESM3_ESM.docx]

Additional Table S2. Presence of reported adverse symptoms across protocols.

|  | 48h Protocol  (n=26) | 24h q12h Protocol  (n=47) | 24h q8h Protocol  (n=2) |
| --- | --- | --- | --- |
| Pruritis | 0 | 1 | 0 |
| Rash | 0 | 1 | 0 |
| Erythema | 0 | 2 | 1 |
| Sedation | 3 | 1 | 0 |
| Respiratory Depression | 1 | 2 | 0 |
| Nausea | 7 | 10 | 1 |
| Worsened Pain | 9 | 11 | 0 |
| Agitation | 9 | 18 | 0 |
| Diarrhea | 5 | 4 | 0 |
| Constipation | 1 | 2 | 0 |
| Insomnia | 4 | 4 | 0 |
| Resulted in drop-out | 1 | 4 | 0 |
